# Bremsstrahlung spectrum data for thick Al, Ti, Zr, Mo, and W targets by keV electron impact

**DOI:** 10.1016/j.dib.2018.01.111

**Published:** 2018-02-06

**Authors:** Zhu An, Lixia Tian, Jingjun Zhu, Mantian Liu

**Affiliations:** a Key Laboratory of Radiation Physics and Technology of Ministry of Education, Institute of Nuclear Science and Technology, Sichuan University, Chengdu 610064, China; b East China University of Technology, Nanchang 330013, China

**Keywords:** Electron impact, Bremsstrahlung, Thick target

## Abstract

The data presented in this paper are related to the research paper entitled “Bremsstrahlung spectra produced by kilovolt electron impact on thick targets” [1]. The dataset includes our measured bremsstrahlung spectra on Al, Ti, Zr, Mo, and W thick targets at 5, 10, 15, 20, and 25 keV electron impact. In this paper we present the experimental method and make the dataset publicly available to enable extended analyses or reuse. The dataset is available on mendeley data public repository at http://dx.doi.org/10.17632/5zx3459bj3.1

**Specifications Table**

**Table:** 

Subject area	Physics
More specific subject area	Atomic Physics
Type of data	Text file
How data was acquired	Data were taken using an ORTEC Si(Li) X-ray detector (Model: SLP-04160)
Data format	Raw, analyzed
Experimental factors	The surfaces of thick Al, Ti, Zr, Mo and W targets were polished.
Experimental features	The bremsstrahlung spectra on Al, Ti, Zr, Mo, and W thick targets at 5, 10, 15, 20, and 25 keV electron impact were measured. The incident electron beam impacted vertically, and the thick targets were tilted by 45°, the X-ray detector was placed horizontally.
Data source location	
Data accessibility	Data is publicly available on mendeley data public repository with 10.17632/5zx3459bj3.1 doi, at http://dx.doi.org/10.17632/5zx3459bj3.1
Related research article	Lixia Tian, Jingjun Zhu, Mantian Liu, Zhu An, Bremsstrahlung spectra produced by kilovolt electron impact on thick targets, Nuclear Instruments and Methods in Physics Research Section B: Beam Interactions with Materials and Atoms 267 (2009) 3495–3499.

**Value of the data**

• These data can be used to compare with Monte Carlo simulations and analytical models for thick-target bremsstrahlung.
• These data can be used to examine bremsstrahlung theory and promote its development.
• These data can be used in some practical applications related to electron-solid interactions.

## 1 Data

The dataset includes 25 text files for our measured bremsstrahlung spectra on Al, Ti, Zr, Mo, and W thick targets at 5, 10, 15, 20, and 25 keV electron impact. These text files are named by element symbol plus incident electron energy, e.g., Al25.TXT means a bremsstrahlung spectrum for Al thick target and 25 keV incident electron energy. Each text file has two columns which correspond to bremsstrahlung X-ray energy (unit: keV) and bremsstrahlung X-ray absolute intensity (unit: 1/eV/sr/electron), respectively. Please also notice that the bremsstrahlung X-ray absolute intensities in this dataset are multiplied by some factors (i.e., 3, 10, 30, 100 for 10, 15, 20, and 25 keV incident electron energies, respectively), except for the case of 5 keV incident electron energy.

## 2 Experimental design, materials and methods

The surfaces of thick Al, Ti, Zr, Mo and W targets were polished. These thick targets were mounted inside a Faraday cup with a top hole and a side hole. The incident electrons with energies of several ten keV were provided by an electron gun and impacted perpendicularly through the top hole, and the thick targets were tilted at an angle of 45°. The incident electrons were collected by the Faraday cup and was fed into an ORTEC digital current integrator (Model 439, ORTEC, USA), which has an accuracy of less than 1% for the charge measurements. The escape rates of incident and secondary electrons from the Faraday cup were estimated by Monte Carlo simulations.

A Si(Li) X-ray detector (Model SLP-04160, ORTEC, USA) was located at the side hole direction for registering bremsstrahlung X-rays generated from the targets, therefore the X-ray emission angle was 90° with respect to the incident electron direction [2]. The Faraday cup and the Si(Li) X-ray detector were placed in a vacuum chamber [2]. The thickness parameters of the Si(Li) X-ray detector, given by the detector manufacturer, were 12.7 μm for Be-window, 38.6 μg/cm^2^ for Au contact layer, 0.1 μm for Si dead-layer, and 4.21 mm for detector sensitive layer. The detector active diameter was 4 mm and no collimator was used. The energy resolution (full-width at half-maximum) for ^55^Fe 5.9 keV Kα X-rays was 160 eV. The Si(Li) detector's efficiencies were determined by using the ^241^Am and ^137^Cs standard X-ray point sources and based on calculations of using the thickness parameters of the Si(Li) detector given by the detector manufacturer [1].

The experimental bremsstrahlung X-ray spectra in absolute intensity units (i.e., 1/eV/sr/electron) were obtained according to Eq. (5) in Ref. [1]. The estimated total uncertainty, except around the bremsstrahlung endpoints, was about 5–9%, which mainly came from the statistical uncertainty of counts (~2–8%) and the uncertainty of the detection efficiency calibration (~5%).

In addition, based on the calibrated detector's efficiencies, we can deduce the solid angle subtended by the Si(Li) X-ray detector, it was about 0.00335 ± 0.00017, therefore we can estimate that the distance from the detector to the target was about 6.12 ± 0.15 cm. These information may be useful for Monte Carlo simulations.
